# Iron response elements (IREs)-mRNA of Alzheimer's amyloid precursor protein binding to iron regulatory protein (IRP1): a combined molecular docking and spectroscopic approach

**DOI:** 10.1038/s41598-023-32073-x

**Published:** 2023-03-28

**Authors:** Mateen A. Khan, Taj Mohammad, Ajamaluddin Malik, Md. Imtaiyaz Hassan, Artem V. Domashevskiy

**Affiliations:** 1 Department of Life Sciences, College of Science & General Studies, Alfaisal University, Riyadh, Saudi Arabia; 2 Centre for Interdisciplinary Research in Basic Sciences, Jamia Millia Islamia, Jamia Nagar, New Delhi, 110025 India; 3 Department of Biochemistry, Protein Research Laboratory, College of Science, King Saud University, Riyadh, Saudi Arabia; 4 Department of Sciences, John Jay College of Criminal Justice, The City University of New York, New York, NY 10019 USA

**Keywords:** Biochemistry, Biological techniques, Biophysics

## Abstract

The interaction between the stem-loop structure of the Alzheimer's amyloid precursor protein IRE mRNA and iron regulatory protein was examined by employing molecular docking and multi-spectroscopic techniques. A detailed molecular docking analysis of APP IRE mRNA∙IRP1 reveals that 11 residues are involved in hydrogen bonding as the main driving force for the interaction. Fluorescence binding results revealed a strong interaction between APP IRE mRNA and IRP1 with a binding affinity and an average binding sites of 31.3 × 10^6^ M^−1^ and 1.0, respectively. Addition of Fe^2+^(anaerobic) showed a decreased (3.3-fold) binding affinity of APP mRNA∙IRP1. Further, thermodynamic parameters of APP mRNA∙IRP1 interactions were an enthalpy-driven and entropy-favored event, with a large negative Δ*H* (–25.7 ± 2.5 kJ/mol) and a positive Δ*S* (65.0 ± 3.7 J/mol·K). A negative ΔH value for the complex formation suggested the contribution of hydrogen bonds and van der Waals forces. The addition of iron increased the enthalpic contribution by 38% and decreased the entropic influence by 97%. Furthermore, the stopped-flow kinetics of APP IRE mRNA∙IRP1 also confirmed the complex formation, having the rate of association (*k*_on_) and the rate of dissociation (*k*_off_) as 341 μM^−1^ s^−1^, and 11 s^−1^, respectively. The addition of Fe^2+^ has decreased the rate of association (*k*_on_) by ~ three-fold, whereas the rate of dissociation (*k*_off_) has increased by ~ two-fold. The activation energy for APP mRNA∙IRP1 complex was 52.5 ± 2.1 kJ/mol. The addition of Fe^2+^ changed appreciably the activation energy for the binding of APP mRNA with IRP1. Moreover, circular dichroism spectroscopy has confirmed further the APP mRNA∙IRP1 complex formation and IRP1 secondary structure change with the addition of APP mRNA. In the interaction between APP mRNA and IRP1, iron promotes structural changes in the APP IRE mRNA∙IRP1 complexes by changing the number of hydrogen bonds and promoting a conformational change in the IRP1 structure when it is bound to the APP IRE mRNA. It further illustrates how IRE stem-loop structure influences selectively the thermodynamics and kinetics of these protein-RNA interactions.

## Introduction

Alzheimer's disease (AD) is a neurodegenerative disorder that onsets people over 65^1^. The disease is characterized by the formation of abundant extracellular plaques, comprising of extracellular amyloid β-peptides senile plaques, intracellular Tau protein neurofibrillary tangles, and an elevated brain iron concentration^2,3^. Iron plays several physiological roles in mitochondrial functions and energy generation in the brain, myelination of neurons, and neurotransmitter synthesis^4^. A surplus of iron in the brain can lead to the pathological factor that drives Alzheimer's and Parkinson's (PD) diseases, as well as other disorders of the Central Nervous System (CNS)^5^. Rogers et al.^6^ found a direct link between iron homeostasis and the AD pathogenesis. Iron levels in the brain influence APP RNA expression in astrocytes^7^ and neuroblastoma cells^6^. The 5'-untranslated region (5'-UTR) of the amyloid precursor protein (APP) transcript includes an IRE within its structure. The regulatory mechanism of APP expression displays similarity to the role of iron in the translation of ferritin mRNA that binds to IREs found in the 5'-UTR of ferritin transcript. As demonstrated through other iron-associated proteins, having an IRE stem loop in the APP transcript suggests this protein's role in iron homeostasis. The APP 5'-UTR codes for a functional iron-responsive element (IRE) RNA stem-loop that represents a likely target for modulating APP expression.

In AD, the poor ability to manage iron binding affects interactions of iron-regulatory proteins, the IRPs, with IREs^8^, leading to implications in the expression of IRE-controlled genes. The iron-responsive elements (IREs) control the posttranscriptional regulation of intracellular iron homeostasis. The IRE mRNA stem-loop structure may be a critical site that causes the misregulation of these key proteins during Alzheimer's. The absence of IRP2 relates to the perturbed metabolism of iron, translation of ferritin RNA, and transferrin receptor (TfR) transcript stability in both the gut mucosa and the CNS^9^. Alzheimer's amyloid precursor protein (APP) mRNA codes for a functional IRE in its 5'-UTR, and this stem-loop structure controls iron-dependent APP synthesis^6,10^. The intracellular iron levels influenced the APP 5'-UTR in a pattern that reflects iron-dependent regulation of intracellular APP synthesis. IRPs are proteins that interact with RNA; IRPs interaction with IRE controls the stability of TfR mRNA and translation of ferritin mRNA. Interaction of IRPs with IREs (specific non-coding sequences within the untranslated, UTR, regions of mRNA transcripts) controls iron metabolism^11^. IREs fold into stem-loop structures comprising 30-nt long RNA motifs with the CAGUGN sequence^11,12^. IREs can be found in both the 3'- and 5'-UTR of the target mRNA^12^. Iron increases rates of iron regulatory protein synthesis in animals by promoting mRNA∙ribosome binding. Labile iron in cells is considered to be ferrous^13^. Only Fe^2+^ has physiological effects on protein biosynthesis directed by riboregulatory iron responsive element mRNA. This riboregulatory structure is also found in mRNA for proteins of iron traffic^14^, nervous system^15^, and cell cycle^16^. Cellular iron levels either stabilize or destabilize the IRP1·IRE mRNA complex formation. High iron accumulation in the brain tissues leads to the IRP1·IRE mRNA signaling pathway irregularities, thus contributing to protein aggregation, neural loss, and progression of AD and PD.

The amyloid toxicity and the APP transcript are subject to regulation by cellular iron level. The APP mRNA encodes the IRE stem-loop containing the CAGA box. The CAGA box is positioned within the 146-nt 5'-UTR of APP mRNA that binds to the IRP1^15^. IRPs control the steady state of iron by influencing the mRNA translation and turn-over of the APP, ferritin, transferrin receptor, and other iron-associated proteins^17^. Fluctuation in cellular iron levels can influence the binding of IRP1 to the IRE mRNA stem- loop, affecting APP, transferrin and ferritin homoeostasis^18^.

Metal ions are abundant at the synapse, and the amyloid proteins affect metal homeostasis in neurons. Reports suggest that disrupting the metal steady state in neurons may lead to the progression of neurodegenerative disease^19,20^. Such crucial microelements as iron (Fe), zinc (Zn), and copper (Cu) play pivotal roles in different brain functions, including neuronal myelination, signal transmission, neurotransmitter synthesis, and protection against reactive oxygen species (ROS). Shortage in these neuro-metals impairs brain functions, causing learning and memory disorders. Iron is the most abundant neuro-metal in the brain. Numerous cellular processes, including mitochondrial energy transfer, oxidative phosphorylation, neuronal myelination and neurotransmitter synthesis, depend on iron as a cofactor^21^. APP regulates iron efflux from cells by binding to an iron transporter ferroportin^22^.

Furthermore, through binding to iron regulatory proteins, iron regulates the expression of proteins containing the iron response element (IRE) sequence^23^. An IRE domain of APP transcript is analogous to that of ferritin and other iron-binding proteins^6^. Hence, APP expression can be controlled via the regulation of metal ion levels in neurons by APP. The binding of IRE mRNA requires IRP1 to undergo a vital conformational change, as observed from the interactions between cytosolic aconitase and IRE-binding protein, forcing substantial domain shifts and conformational changes to the procedure of RNA-binding pockets. Circular dichroism (CD) spectroscopy and X-ray crystallography revealed these IRP1 structural conformational changes after binding to IRE^24,25^. Similar methods were employed to elucidate structural changes during eIFs (eukaryotic translation initiation factors) binding to IRE mRNA and mRNA cap moiety^26,27^. It is unknown what changes APP IRE mRNA-binding induces in the structure of IRP1. The APP IRE mRNA·IRP1 complex's strength and stability are driven by entropy and enthalpy, producing favorable free energy. This necessitates a thermodynamic insight. In our study, we consider temperature effects and iron concentrations on the equilibrium and kinetic rates of APP IRE mRNA binding to IRP1. Thermodynamic studies showed a significant change in free energy and enthalpy for the APP IRE mRNA·IRP1 complex formation, suggesting changes in hydrogen bonding with overall conformational changes during complex formation. To better understand the APP IRE mRNA·IRP1 binding changes, we investigated the kinetics of APP IRE interaction with IRP1. Iron prompted significant changes in the activation energy for the APP IRE mRNA·IRP1 complex. A molecular docking study was performed to get deeper insights into the structural basis of IRP1 and APP IRE mRNA oligonucleotide binding by exploring their interactions.

## Results

### Molecular docking analysis of APP IRE mRNA and IRP1 binding

**Figure 1:**
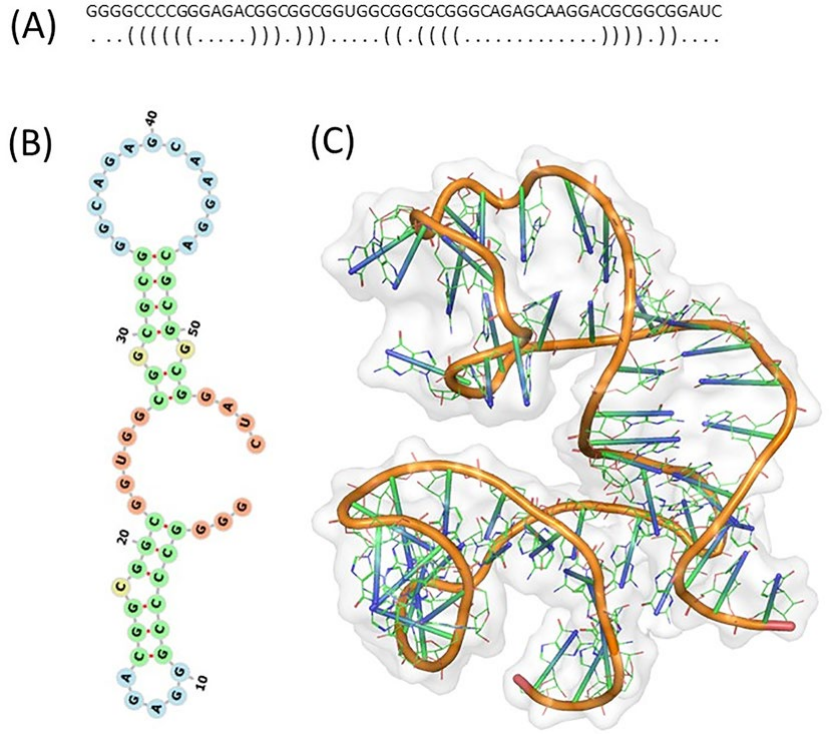
APP IRE mRNA sequence and dot-bracket notation (**A**) for secondary structure (**B**), and tertiary (**C**) structure prediction.

RNA function depends on its structural conformation, and its functional ability to bind to a protein molecule^28^. Prediction of the tertiary structure is important; it aids in identification of the nucleotide residues, responsible for the IRE mRNA conformation and RNA–protein interaction. Here we performed predictions of secondary and tertiary structures, as well as docking models for RNA and RNA/protein complex. APP IRE mRNA (57-nt) sequence was used for the folded RNA secondary structure predictions. The sequence and the dot-bracket notation were used as input to predict the secondary structure of the APP IRE mRNA (Fig. 1A). CentroidFold method was employed to get the dot-bracket format of the RNA sequence. Figure 1B shows the predicted secondary structure of the APP IRE mRNA. RNAFold WebServer was used to predict folding of the RNA structure. The prediction of APP IRE mRNA secondary structure is dominated by base-paired stems and hairpin loops. The minimum free energy (Δ*G*) was − 20.30 kcal/mol. The predicted secondary structure of APP IRE mRNA bears similarities to the ferritin IRE mRNA stem-loop structure^29^.

**Figure 2:**
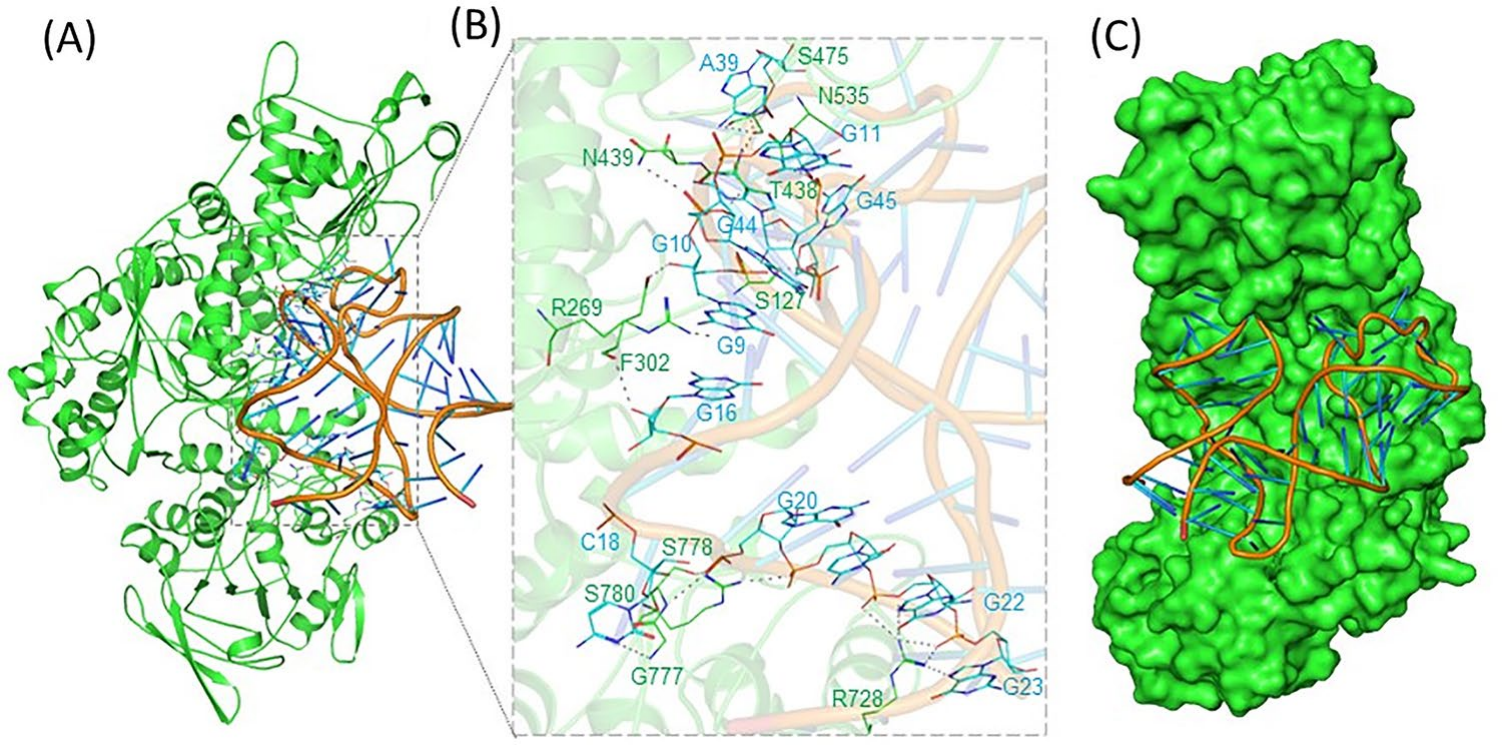
Molecular interaction of APP IRE mRNA with IRP1. (**A**) Cartoon diagram of IRP1 with APP IRE mRNA showing superimposition of docked IRE mRNA (light brown ribbon) with IRP1 (green ribbon) complex. (**B**) Zoomed cartoon diagram of IRP1 showing interactions between residues of IRP1 and IRE mRNA. (**C**) Binding cavity of IRP1 occupied by IRE mRNA.

The tertiary structure was built using the dot-bracket format of the secondary structure of APP IRE mRNA by RNAComposer^30^. The molecular docking of the APP IRE mRNA with the IRP1 protein molecules was simulated on the HDOCK web server^31^. This computational tool allowed identification of residues and the interaction types between the RNA and protein. Figure 2 illustrates a detailed binding pattern of the selected docked model of IRP1 and APP IRE mRNA oligonucleotide interaction. The docking of IRP1 and APP IRE mRNA oligonucleotide predicts several possible docking models with appreciable binding affinities; a single model with the best docking score was used for further analysis. The top hit models were selected out of all possible models based on the interaction score. APP IRE mRNA oligonucleotide binding to IRP1 had an appreciable docking score, as calculated as − 364.09. This docking score was calculated by HDOCK inbuilt knowledge-based iterative scoring function ITScorePP. A more negative docking score means a more possible binding model. Given that the protein-RNA complexes in the PDB normally have a docking score of around − 200 or better. In our case, the predicted docking score suggested an appealing binding of APP IRE mRNA oligonucleotide towards IRP1.

Further, a detailed interaction analysis of the selected docked complex was carried out to explore the binding mode of the IRP1∙APP IRE mRNA oligonucleotide interaction. Figure 2C shows the APP IRE mRNA in the binding pocket of the IRP1 protein. APP IRE mRNA bound IRP1 in a cleft. APP IRE mRNA oligonucleotide interacts with a set of functionally active residues of the IRP1 binding site (Fig. 2A). IRP1 is depicted as a cartoon model in pale green, and RNA is shown in light brown in element, ball, and stick models. RNA binds to the several IRP1 residues, specifically the ones that form hydrogen bonds as depicted by black dashes (Fig. 2B). Figure 2B shows that eleven hydrogen bonds stabilize the IRP1∙APP IRE mRNA oligonucleotide complex along with other interactions. Residues Ser127, Arg269, Phe302, Thr438, Asn439, Ser475, Asn535, Arg728, Gly777, Ser778, and Ser780 of IRP1 and the stem-loop oligonucleotide residues of APP IRE mRNA participate in hydrogen bonding interactions during the complex formation. These residues are in direct contact with the bound APP IRE mRNA. APP IRE mRNA oligonucleotide showed a structural complementarity fit with the binding pocket of IRP1 (Fig. 2C). APP IRE mRNA binding appears to fit snugly into the internal pocket of the IRP1 protein.

### Binding analysis of APP IRE mRNA with IRP1

**Figure 3:**
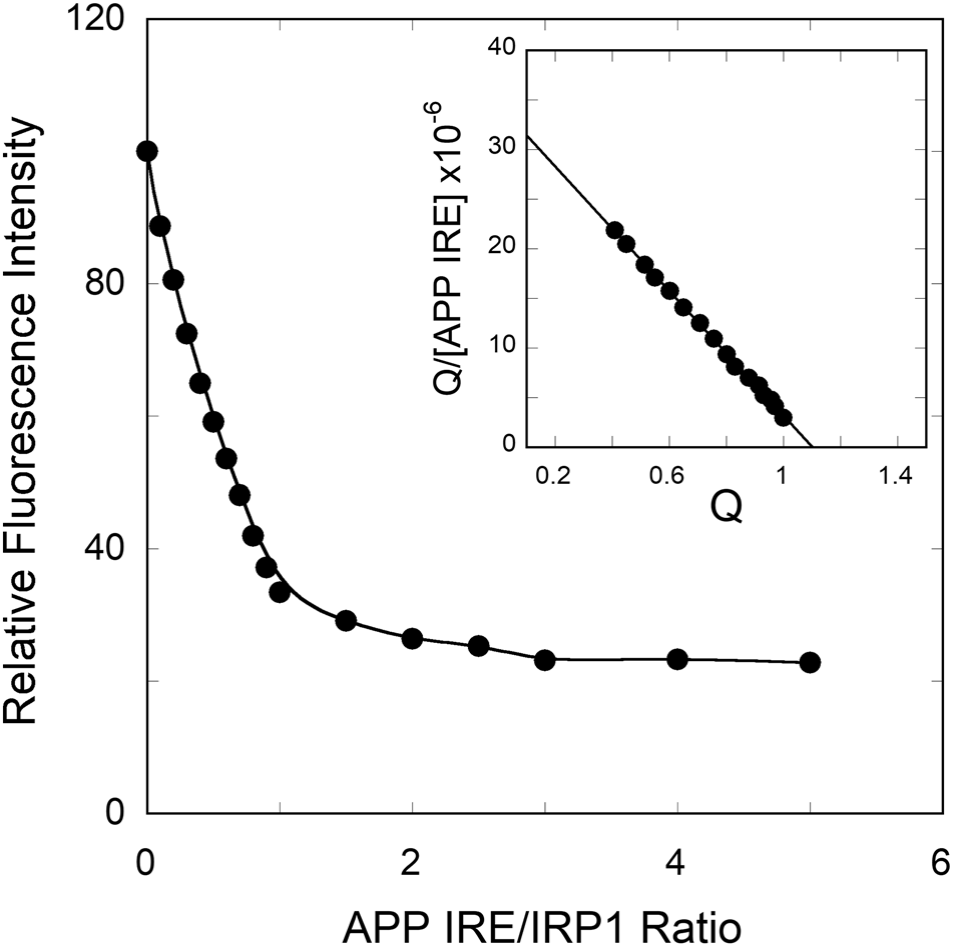
Fluorescence intensity of APP IRE mRNA binding to IRP1 protein was monitored quenching of the intrinsic protein fluorescence at 25 °C. Fluorescence intensity measurements of APP mRNA (—●—) binding to IRP1. For protein titration, the excitation maximum was 280 nm and emission maximum were monitored at 332 nm. The inset shows the corresponding Scatchard analyses of the titration data. The solid lines are fitted theoretical curves.

**Figure 4:**
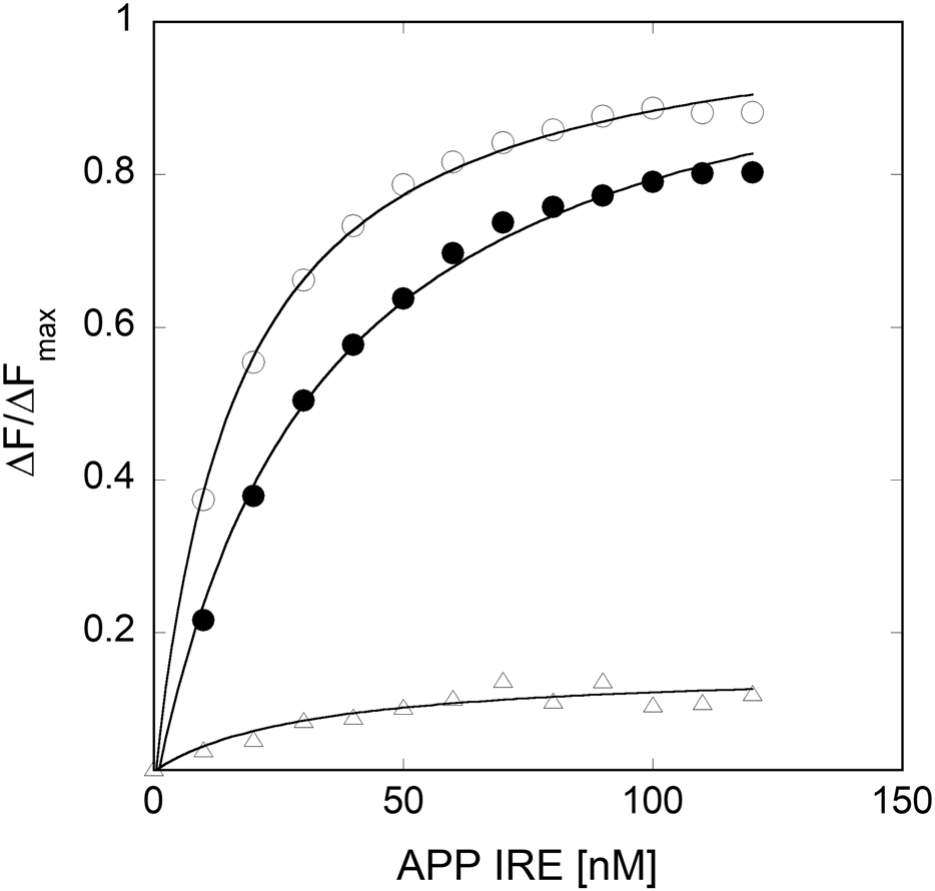
Fluorescence intensity measurements for the binding of APP IRE mRNA to IRP1 protein in the presence of iron (anaerobic). APP mRNA (57-nucleotides) was melted and annealed prior to each titration and incubated with IRP1 protein. 100 nM IRP1 protein was incubated with varying amounts of APP IRE mRNA in the absence (—○—) and presence (—●—) of 50 µM iron. The excitation wavelength was 280 nm and emission wavelength were 332 nm. The solid lines are the fitted curves.

Molecular docking studies for the interaction between IRP1 and APP IRE mRNA were further supported by fluorescence spectroscopy experimental binding studies. To measure the binding affinity of APP mRNA with IRP1 protein, direct fluorescence titration studies were performed as presented in Fig. 3. The quenching of intrinsic protein fluorescence at 334 nm was measured. It was assumed that the amount of protein fluorescence quenching is proportional to the amount of APP mRNA bound to IRP1, suggesting the complex formation. The inset of Fig. 3 shows the corresponding Scatchard plot for the APP IRE mRNA∙IRP1 interactions. The binding affinity (*K*_a_) and the binding capacity (*n*) were determined from the slope and the intercept of the Scatchard plot Q/[IRE] × 10^–6^ *versus* Q. The binding affinity and the binding capacity for the interaction of APP mRNA with IRP1 were 31.3 × 10^6^ M^−1^ and 1.0, respectively. The inset shows the results of the corresponding Scatchard plot, indicating a single binding site for the APP mRNA binding to IRP1. The *K*_D_ value for APP mRNA binding to IRP1, determined here by Scatchard analysis, agree well with the value obtained using a non-linear least squares analysis (*K*_D_ = 32.0 nM) (Fig. 4). The equilibrium constants are reported either as the dissociation constants (*K*_D_) or the association constants (*K*_a_ = 1/*K*_D_). The results obtained by two-independent data analysis methods are in good agreement.

We have reported previously^32^ that iron metal ions destabilize the ferritin IRE mRNA·IRP1 complex. Here we show the effect of iron on the binding affinity of stem-loop structure of APP IRE mRNA to IRP1. Anaerobic addition of Fe^2+^ caused the binding affinity of APP mRNA for IRP1 to decrease about threefold (IRP1∙APP mRNA-Fe^2+^, *K*_*D*_ = 111.0 nM; IRP1∙APP mRNA, *K*_D_ = 32 nM) at 25 °C. On the other hand, the 30-oligonucleotide stem-loop, derived from yeast 5*S* RNA (a negative control), did not bind IRP1 under the same experimental conditions (Fig. 4), suggesting that IRP1 specifically recognizes the APP mRNA stem-loop, as observed previously for the stem-loop found in ferritin IRE mRNA^32^. These data provide quantitative information to support the specific binding of stem-loop APP IRE mRNA to IRP1.

Furthermore, we measured the temperature-dependent binding affinity of APP mRNA to IRP1 in the absence and presence of iron. Fluorescence titration experiments were performed in the temperature range of 5 °C to 25 °C. Temperature-dependent dissociation constant values are presented in Table 1. Fluorescence studies results at different temperatures revealed that the binding constants (*K*_D_) increased as the temperature elevated from 5 °C (*K*_D_ = 8.1 ± 0.3 nM) to 25 °C (*K*_D_ = 32 ± 0.6 nM) in the absence of iron. Lower temperature stabilizes APP mRNA·IRP1 complex. Analysis of the fluorescence data reveals that the *K*_D_ value of the APP mRNA∙IRP1 complex at 25 °C was significantly higher than at 5 °C (Table 1). As the temperature elevated from 5 °C to 25 °C, the dissociation constant for the IRP1·APP mRNA-Fe^2+^ complex increased from 19.3 ± 0.6 nM to 111 ± 2.8 nM (Table 1). Addition of iron (50 μM Fe^2+^, anaerobic) shows an increase in the *K*_D_ values of APP mRNA∙IRP1 at the range of temperature studied. The binding data showed that at all five temperatures APP mRNA had consistently higher dissociation constant from APP mRNA∙IRP1 complex in the presence of iron (Table 1).

**Table 1:** Temperature-dependent dissociation constants (*K*_D_) for the interaction of APP IRE mRNA with IRP1 in the absence and presence of iron (anaerobic).

Complex	*K*_D_ (nM)				
	5 °C	10 °C	15 °C	20 °C	25 °C
APP IRE mRNA-IRP1	8.1 ± 0.3	14.7 ± 0.2	20.2 ± 0.5	26.1 ± 0.4	32.0 ± 0.6
APP IRE mRNA-IRP1-Fe^2+^	19.3 ± 0.6	38.2 ± 0.5	61.1 ± 2.7	83.4 ± 3.4	111 ± 2.8

### Thermodynamics of APP IREmRNA∙IRP1 binding

**Figure 5:**
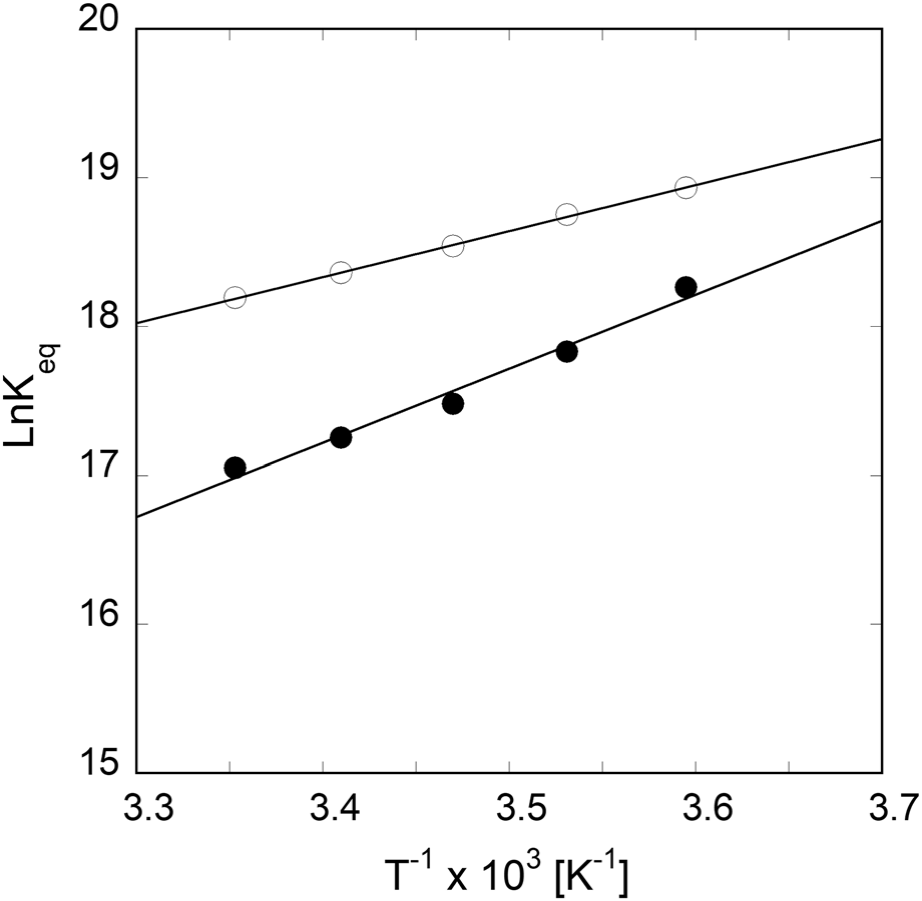
van't Hoff plots for the interaction of APP IRE mRNA with IRP1 protein in the absence and presence of iron. Temperature-dependent binding affinity of IRP1∙APP IRE mRNA (—Ο—) and IRP1∙APP IRE mRNA-Fe^2+^ (—●—) complex.

To further support the mechanism of APP IRE mRNA∙IRP1 interaction, thermodynamic analysis was performed using Eqs. 3 and 4. The temperature dependence of the dissociation constant for the binding of APP mRNA to IRP1 was used to determine the thermodynamic parameters in the absence and presence of iron. The equilibrium data were analyzed by the van't Hoff equation. Figure 5 shows the van't Hoff plots for binding of APP mRNA to IRP1. The thermodynamic parameters of enthalpy (Δ*H*) and entropy (Δ*S*) were obtained from the slope and the intercept of the van't Hoff plot, respectively (Fig. 5). Changes in enthalpy and entropy, and the corresponding free energies for the formation of the complexes determined from the van't Hoff plot are presented in Table 2. Fitting temperature-dependent fluorescence data to the plot yielded the Δ*H* and Δ*S* values of − 25.7 ± 2.5 kJ/mol and 65.0 ± 3.7 J/mol/K, respectively, for APP mRNA∙IRP1. The addition of iron significantly changes the Δ*H* and Δ*S* of binding for the APP mRNA∙IRP1 to − 41.4 ± 3.3 kJ/mol and 2.6 ± 0.2 kJ/mol, respectively (Table 2). The Δ*G* value was calculated at 298 K. Interestingly, the Δ*G* value for the interaction of APP mRNA∙IRP1 complex changed significantly with the addition of iron.

**Table 2:** Thermodynamic parameters of enthalpy (Δ*H*), entropy (Δ*S*), and Gibb’s free energy (Δ*G*) for the interactions of APP IRE mRNA with IRP1.

Complex	Δ*H* (kJ/mol)	Δ*S* (J/mol/K)	Δ*G* (kJ/mol)
APP IRE mRNA·IRP1	− 25.7 ± 2.5	65.0 ± 3.7	− 46.3 ± 3.2
APP IRE mRNA·IRP1_Fe^2+^	− 41.4 ± 3.3	2.6 ± 0.2	− 41.1 ± 3.4

ΔG values were calculated using the equation ΔG = ΔH − TΔS at 25 °C.

We observed that thermodynamic parameters Δ*H* and Δ*G* have negative values, favoring the association of APP mRNA with IRP1. The sign and magnitude of the thermodynamic parameters indicate a particular force's involvement in protein–ligand interaction^33^. The negative Δ*G* values suggest that the complex formation at five different temperatures was feasible, and the binding reaction was spontaneous. The negative Δ*H* value characterized the binding reaction as exothermic. However, a large negative Δ*H* value for the complex formation suggests hydrogen bonds be the dominant forces in the APP IRE mRNA∙IRP1 interaction^33,34^. The free energy for the binding of APP mRNA to IRP1 was − 46.3 ± 3.2 kJ/mol. The addition of Fe^2+^ lowered the binding free energy of APP mRNA binding to IRP1 to about − 5.2 kJ/mol, a typical value for single hydrogen bond or salt bridge formed between APP IRE mRNA and IRP1^35,36^. Change in the Δ*G* of binding for APP mRNA∙IRP1 complexes in the presence of iron, suggests that iron induces conformational changes in APP mRNA⋅IRP1 complexes by changing the number of hydrogen bonds.

### Kinetic analysis of APP IRE mRNA∙IRP1 association

**Figure 6:**
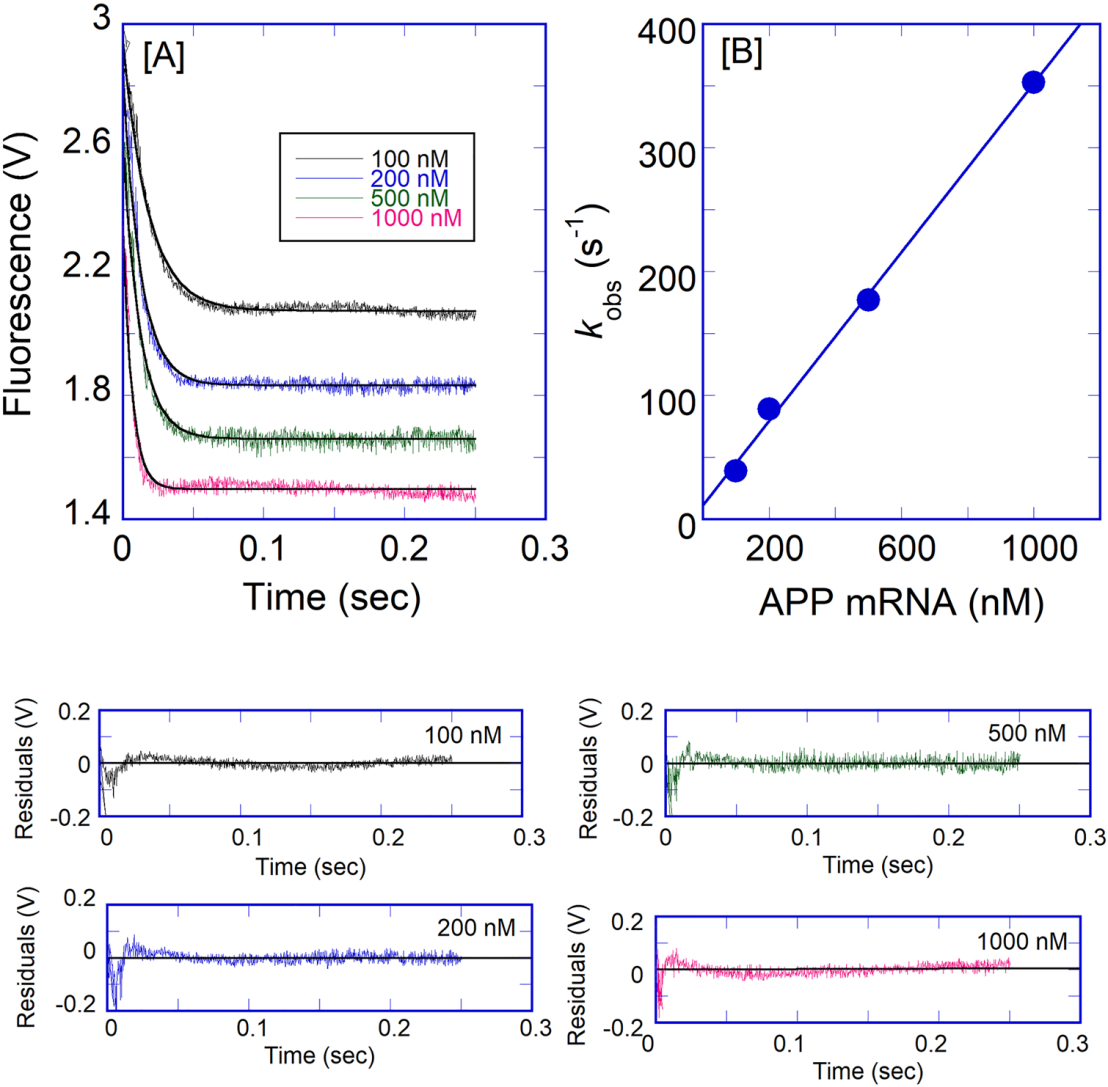
Kinetic rates for the binding of APP IREmRNA to IRP1 protein. (**A**) Typical time course of the intrinsic protein fluorescence intensity of IRP1 (0.1 μM final) decrease caused by binding of APP mRNA at varying concentration (0.1, 0.2, 0.5, and 1 μM final). (**B**) The observed rate constant for the binding of IRP1 to APP mRNA was plotted as a function of increasing concentrations of APP mRNA. The solid line represents the fitted curve for a single exponential function. Residuals for the corresponding fits are shown in the lower panels.

Figure 6 shows representative stopped-flow traces obtained upon rapid mixing of 0.1 μM (final) IRP1 with 0.1–1 μM (final) APP IRE mRNA. The binding reaction of IRP1 was probed over the range of APP mRNA concentrations (Fig. 6A). On the ordinate is the relative voltage, which is proportional to fluorescence intensity. The rapid mixing of IRP1 with APP mRNA resulted in a decrease in fluorescence intensity, which was dependent on the APP mRNA concentration. Data for the binding of IRP1 to APP mRNA were plotted as fluorescence (in volts) *versus* time. The traces followed single-exponential kinetics over all APP IRE mRNA concentrations. Time course data were fitted by non-linear regression analysis. The residuals, representing the deviation between the calculated and experimental data, indicate that the single exponential function fits the points over the entire time course of the measurements. The residuals did not vary over time, nor were they improved by a double-exponential fitting (data not shown). Under pseudo first-order conditions, if the association reaction is a simple one-step process, the observed rate is predicted to vary linearly with the APP mRNA concentrations. The reaction rate concentration-dependence also allowed us to differentiate whether a single bimolecular binding or a more intricate mechanism is in place (e.g., fast association followed by a conformational change). The equation below, describes a one-step reaction mechanism that fits our experimental results (where *k*_on_ and *k*_off_ are the association and dissociation rate constants for the binding of APP mRNA to IRP1):

1 $${\text{APP}}\;{\text{mRNA}} + {\text{IRP1}}\mathop{\longrightarrow}\limits_{{k_{{{\text{off}}}} }}^{{k_{{{\text{on}}}} }}{\text{APPmmRNA}} \cdot {\text{IRP1}}$$

The observed rate constant (*k*_obs_) is predicted to be a linear function of the APP mRNA concentration, as shown in the equation:

2 $$k_{{{\text{obs}}}} = k_{{{\text{on}}}} [{\text{APP}}\,{\text{mRNA}}] + k_{{{\text{off}}}}$$

where the rates of fluorescence intensity change for IRP1 decreased with the increasing of APP mRNA concentration (Fig. 6A). A plot of *k*_obs_ *versus* [APP mRNA] was linear (Fig. 6B), indicating that the association of IRP1 with APP mRNA follows a simple one-step binding mechanism. The slope and the *y*-intercept are *k*_on_ and *k*_off_, respectively^37^. The *k*_on_ and *k*_off_ values from Fig. 6B were (341 ± 15) × 10^6^ M^−1^ s^−1^ and 11 ± 0.4 s^−1^, respectively.

**Figure 7:**
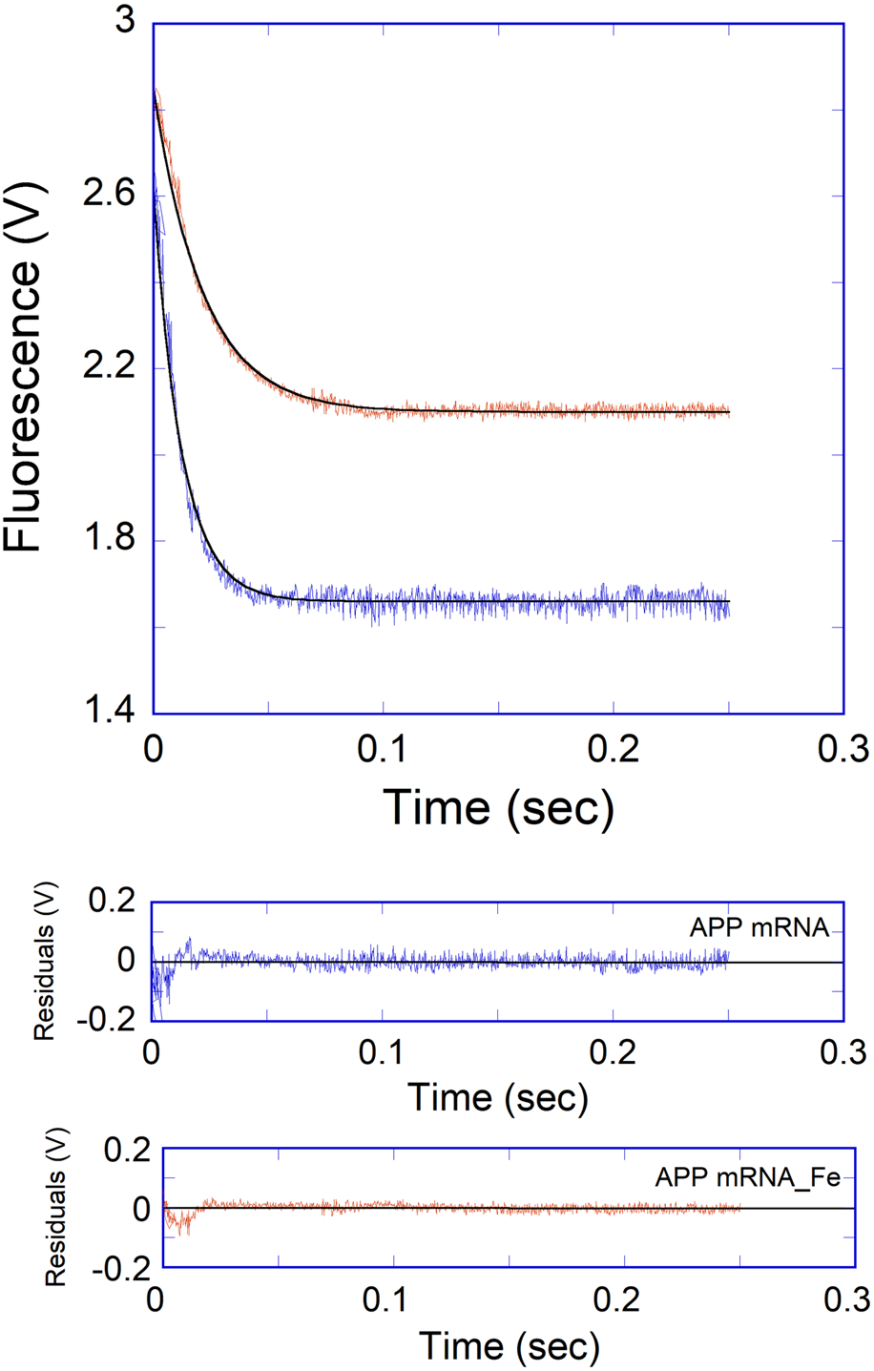
Iron decreases the kinetic rates for the binding of APP IRE mRNA to IRP1. Representative kinetic data show the time-dependent decrease in fluorescence intensity caused by binding of APP mRNA and APP mRNA-Fe^2+^ with IRP1. APP mRNA and IRP1 concentration were 500 nM and 100 nM (final), and the Fe^2+^ concentration was 50 μM (final). The solid line represents the fitted curve for a single-exponential function. The residual for the exponential fits is shown in lower panels.

**Figure 8:**
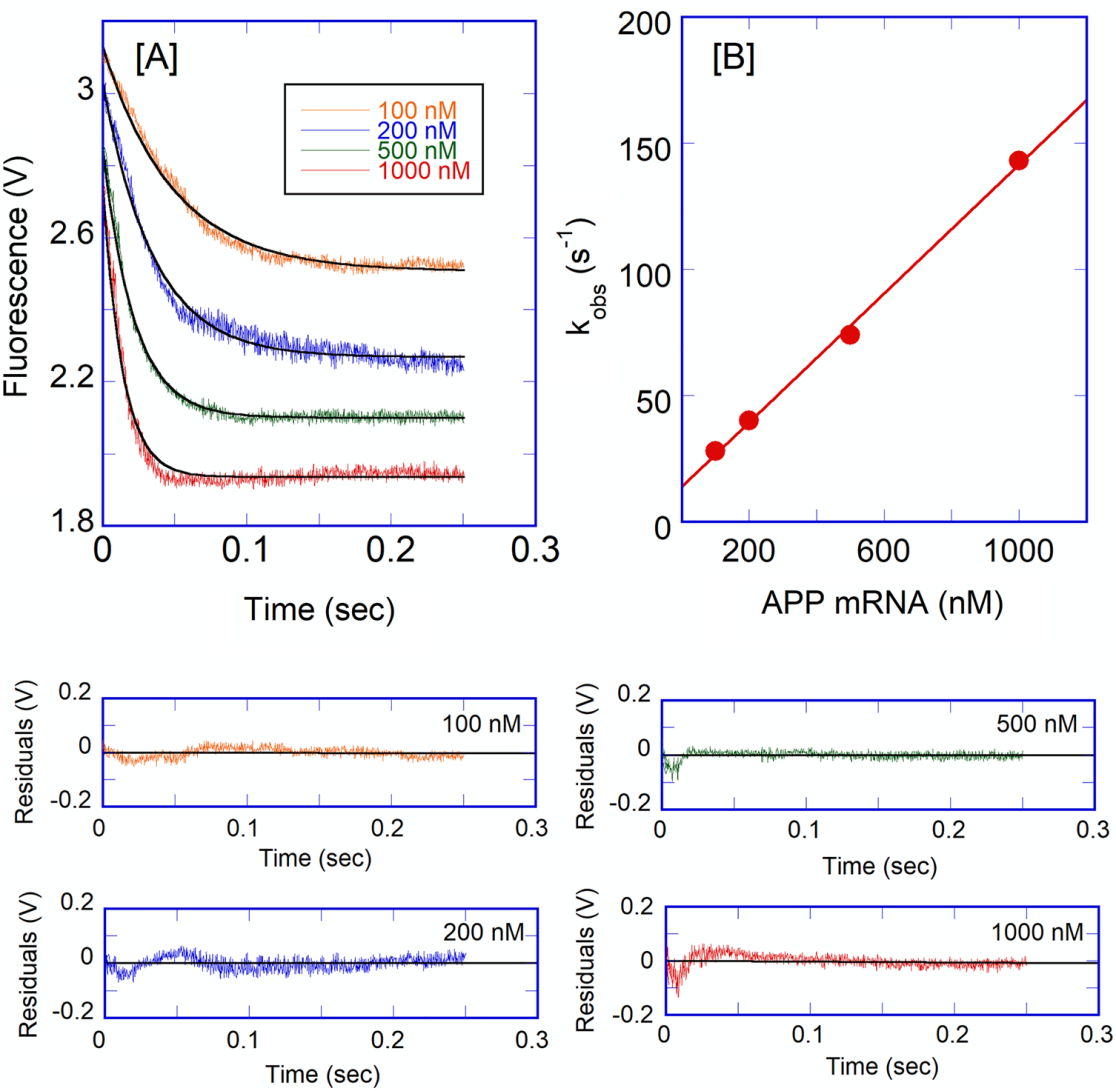
Kinetic rates for the binding of APP IREmRNA to IRP1 protein in the presence of iron. (**A**) Kinetic trace for the binding of IRP1 (0.1 μM final) at varying concentration of APP mRNA (0.1, 0.2, 0.5, and 1 μM final) in the presence of 50 μM Fe^2+^. (**B**) The observed rate constant for the binding of IRP1 with APP mRNA in the presence of iron was plotted as a function of increasing concentrations of APP mRNA. Data points in the plots of *k*_obs_ *versus* APP mRNA concentration were obtained from the three independent experiments, and the average value of the experimental data was reported. The solid line represents the fitted curve for a single exponential function. Residuals for the corresponding fits are shown in the lower panels.

Iron affected the binding affinity of APP IRE mRNA to IRP1. To further understand the mechanism of this reaction, the effects of iron on the kinetics of the reaction were investigated. 50 μM Fe^2+^ to APP IRE mRNA under complex formation conditions with rapid mixing of IRP1, the observed rate constant decreased significantly (Fig. 7). The traces followed single-exponential kinetics over the APP mRNA concentrations. The residuals did not vary over time, nor were they reduced by a double-exponential fit (not shown). The addition of iron decreased the association rates of APP mRNA with IRP1. Kinetic plots for the binding of APP mRNA with IRP1 in the presence of 50 μM iron are shown in Fig. 8A. Plots of the observed rate constant *versus* APP mRNA concentrations are shown in Fig. 8B for IRP1 in the presence of iron. The association rate constant (*k*_on_) for the binding of APP mRNA to IRP1 in the presence of iron was (127 ± 5) × 10^6^ M^−1^ s^−1^, whereas the dissociation rate constant (*k*_off_) was 16 ± 0.5 s^−1^. The addition of iron lowered the association rate constant (*k*_on_) by about 2.7-fold, whereas it increased the dissociation rate constant (*k*_off_) by about 1.4-fold for the binding of IRP1 to APP mRNA.

**Figure 9:**
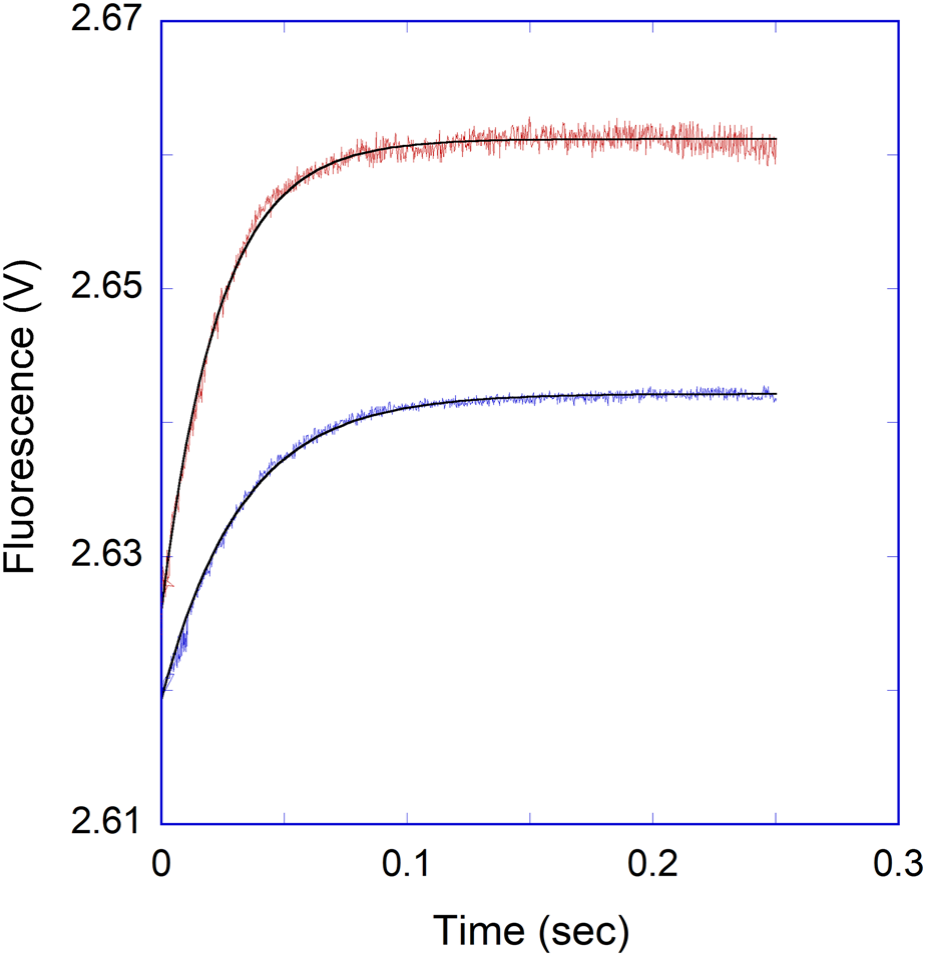
Iron decreases APP IRE mRNA·IRP1 binding by changing *k*_on_ and *k*_off_. APP mRNA·IRP1 complex was diluted in the stopped-flow cell with an equal volume of the titration buffer alone, curve (**A**) and 50 μM Fe^2+^ in the titration buffer, curve (**B**). Traces were best fit with the single-exponential function. The concentration of APP mRNA was 1 μM, and IRP1 was 0.1 μM after mixing. Data points were averaged from three independent experiments. The *k*_off_ value was obtained from the *K*_eq_ values and using *K*_d_ = *k*_off_/*k*_on_.

The effect of iron on the dissociation of the pre-formed APP mRNA·IRP1 complex was also investigated through the measurement of fluorescence intensity of the relaxation reaction when equal volumes of APP mRNA·IRP1 were rapidly mixed with either buffer alone or with 50 μM Fe^2+^ in the solution buffer. The kinetic traces of the dissociation reactions initiated by the twofold dilution followed single-exponential kinetics (Fig. 9). The dissociation rates for the APP mRNA·IRP1 complex increased twofold (*k*_off_ = 18 ± 0.4 s^−1^) with an addition of 50 μM Fe^2+^ in a buffer as compared to the buffer alone (*k*_off_ = 10 ± 0.3 s^−1^). The dissociation rates for the APP mRNA·IRP1 complexes (obtained by diluting either with buffer alone or buffer containing 50 μM Fe^2+^) were similar to the dissociation rate determined from concentration-dependent reaction kinetics (*k*_off_ = 11 ± 0.4 s^−1^ for APP mRNA·IRP1 and 16 ± 0.5 s^−1^ for IRP1∙APP mRNA∙Fe^2+^ complex). These results suggest that iron selectively increases the dissociation rates of the APP mRNA·IRP1 complexes.

**Figure 10:**
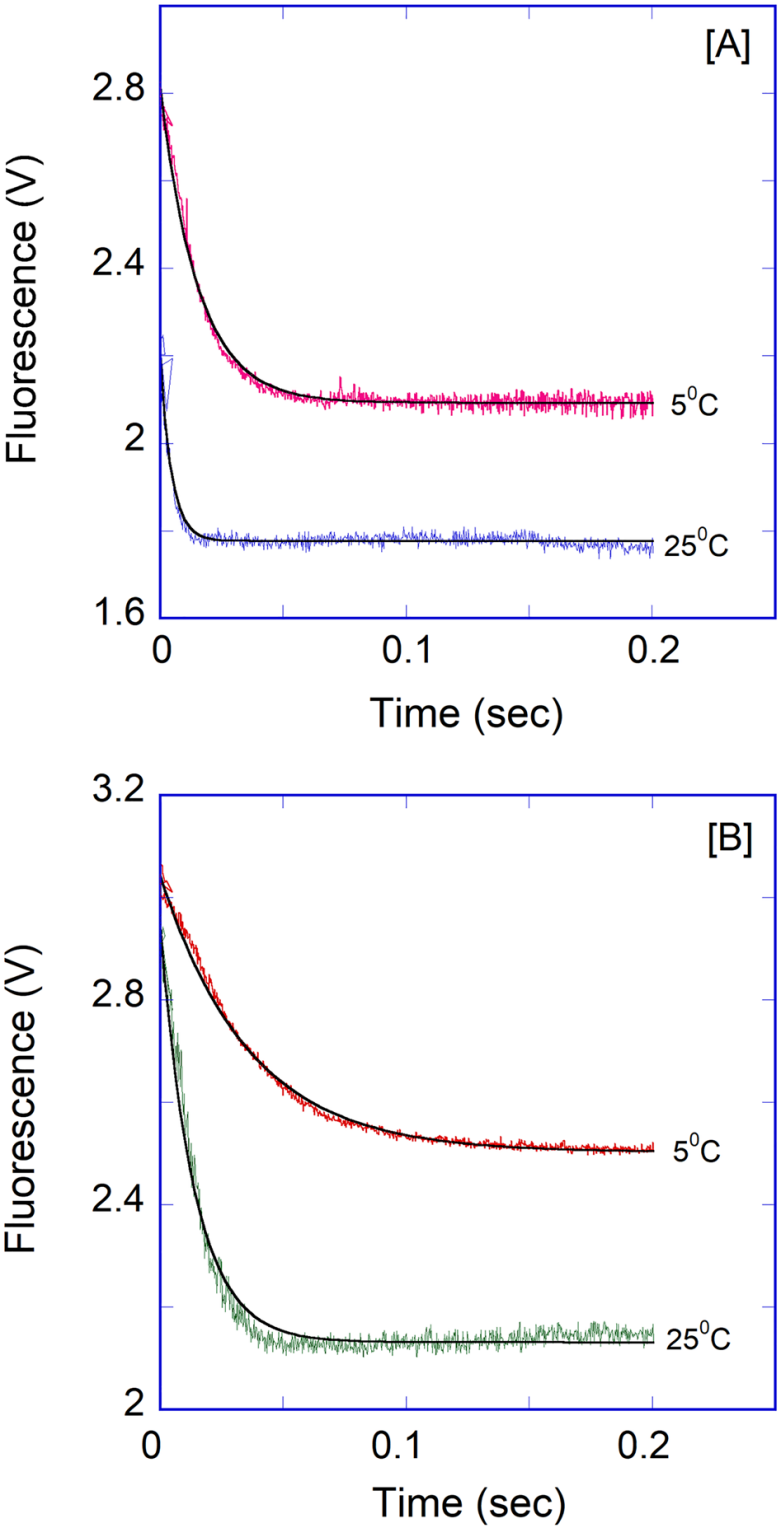
Representative temperature-dependent time course for the interaction of APP IREmRNA with IRP1. Kinetic data show the time-dependent decrease in fluorescence values after rapid mixing of (**A**) APP mRNA and (**B**) APP mRNA∙Fe^2+^ with IRP1 protein at 5 °C and 25 °C. APP mRNA and IRP1 concentrations were 1 μM (final) and 0.1 μM (final). Concentration of Fe^2+^ was 50 μM. The solid line represents the fitted curve for a single-exponential function. The experimental conditions are described in the Methods section.

**Figure 11:**
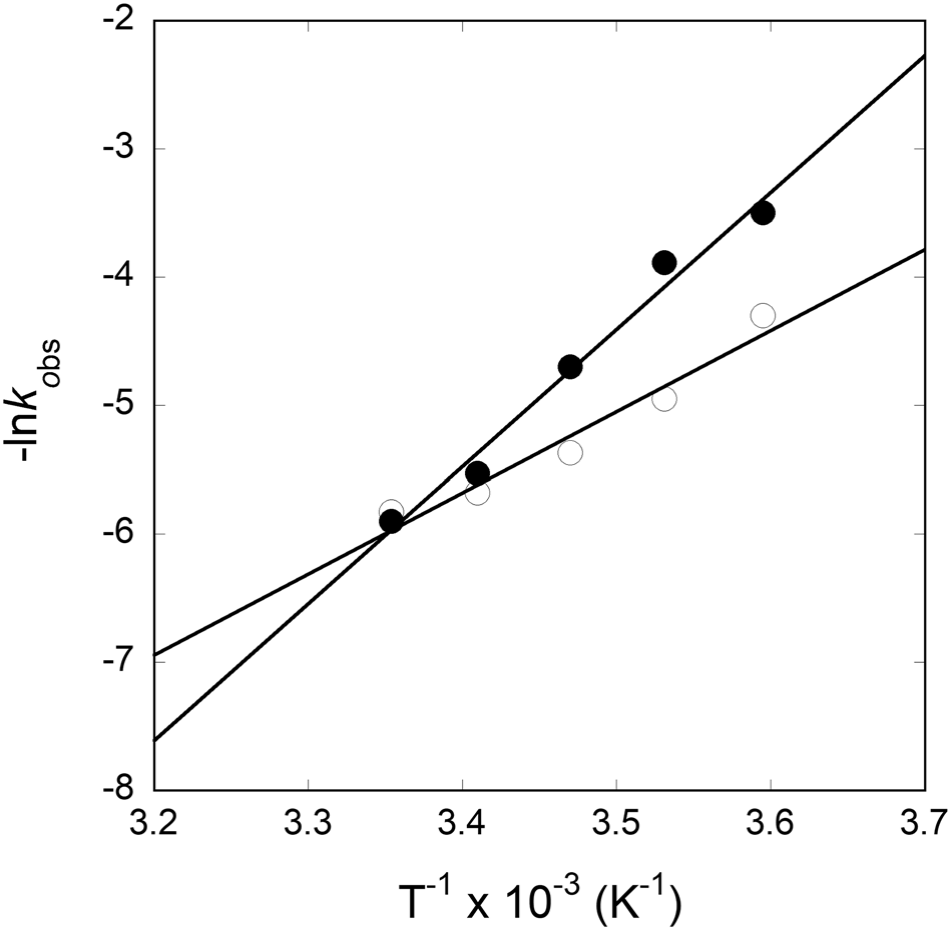
Arrhenius plots for determining the interaction between APP IREmRNA and IRP1 with and without iron. Temperature-dependent observed rate constant of IRP1·APP IRE mRNA (—Ο—) and IRP1·APP IRE mRNA-Fe^2+^ (—●—) complex was used for the plots. The final concentrations of APP mRNA, IRP1, and Fe^2+^ were 500 nM, 100 nM, and 50 μM, respectively.

We further examined the temperature-dependent kinetic rates for the binding of APP IRE mRNA to IRP1 in the absence and presence of iron. The observed rate constants for APP mRNA binding to IRP1 at 5, 10, 15, 20, and 25 °C in the absence and presence of iron are presented in Table 3. The *k*_obs_ for APP mRNA binding to IRP1 at different temperatures were calculated from the data sets collected at each temperature, as shown in the example in Fig. 10. A non-linear regression analysis fitted kinetic traces following a single exponential fit at all five temperatures. The observed rate constants for the interaction of APP mRNA with IRP1 in the absence and presence of iron increased with an increase in temperature (Fig. 10, Table 3). The kinetic data showed that the APP mRNA∙IRP1 complex binding rate at 25 °C (*k*_obs_ = 341 s^−1^) was ~ five-fold faster than at 5 °C (*k*_obs_ = 74 s^−1^). Figure 10B shows the representative kinetic trace for the binding of APP mRNA to IRP1 after adding iron. The kinetic rate constant for the IRP1∙APP mRNA-Fe^2+^ complex increased with an increased temperature from 5 °C to 25 °C. The kinetic data showed that the observed kinetic rate of IRP1∙APP mRNA-Fe^2+^ complex at 25 °C was much faster than at 5 °C. Analysis of the temperature-dependent kinetic data showed that the addition of iron lowered the kinetic rates for binding the APP mRNA∙IRP1 complex. To determine the activation energies of the APP mRNA binding to IRP1 in the absence and presence of iron, the temperature-dependent observed rate constant values were used to construct the Arrhenius plots. The activation energy was calculated from the slope of the fitted linear line of ln *k versus* 1/*T* (Kelvin) (Fig. 11). The activation energy for APP mRNA binding to IRP1, determined from the temperature-dependent rate constants, was 52.5 ± 2.1 kJ/mol. The addition of iron to the APP mRNA∙IRP1 complex increased the activation energy to 86.0 ± 3.2 kJ/mol. These data show a large change in the activation energy for the APP mRNA∙IRP1 complex with the addition of iron. Change in the activation energy for the APP mRNA binding to IRP1 after the addition of iron, suggests that iron induces conformational changes in the APP mRNA∙IRP1 complex, triggering a release of IRP1 repressor protein, and allowing translation initiation factor binding. Subsequently, this leads to an increase in protein synthesis.

**Table 3:** Temperature-dependent observed rate constants of APP IRE-mRNA binding to IRP1 with and without Fe^2+^.

Complex	*k*_obs_ (s^−1^)					*E*_a_
	5 °C	10 °C	15 °C	20 °C	25 °C	kJ/mol
APP IRE mRNA·IRP1	74 ± 4.3	141 ± 6.2	215 ± 9.5	292 ± 8.4	341 ± 9.6	52.5 ± 2.1
APP IRE mRNA·IRP1-Fe^2+^	31 ± 1.6	49 ± 2.5	110 ± 3.7	252 ± 9	365 ± 14	86.0 ± 3.2

### Secondary structure characterization by far-UV CD measurements

**Figure 12:**
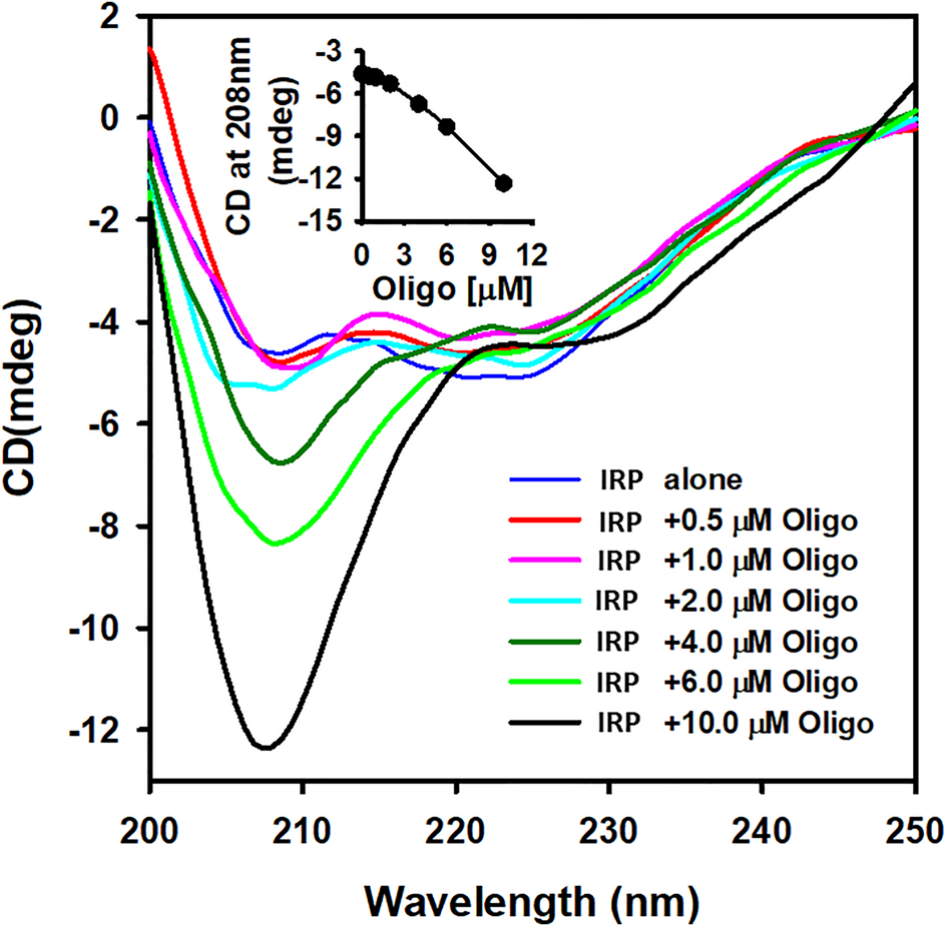
CD spectra of iron regulatory protein (IRP1) as a function of Alzheimer's amyloid precursor protein (APP) IRE mRNA. The far-UV CD spectra were obtained using IRP1 protein concentration of 0.1 µM with addition of varying amounts of APP IRE mRNA (0 – 10 µM).

CD spectroscopy in the far-UV region provide an essential information about the conformational properties of proteins in solution. Changes in the far UV-CD protein spectra correspond to the change in the secondary structure of protein. In this study, we examined the change in the secondary structure of native IRP1 protein and IRP1 upon binding to APP IRE mRNA by measuring the far-UV (200–250 nm) CD spectra at 25 °C. Figure 12 shows the far-UV CD spectra of the free IRP1 (0.1 μM) protein and after the addition of different concentrations of the APP IRE mRNA (0–10 μM). The far-UV region CD measurements present data that has been useful in examining the secondary structure changes of proteins, for example, an amount of α-helix, β-sheet, β-turn, and random coil^38,39^. It can be seen from the spectra that the free IRP1 shows a single negative peak at 208 nm, characteristic of a typical α-helix in the far-UV region and a little peak at 218 nm characteristic of β-sheet conformation in protein. The addition of increasing amounts of APP mRNA showed an increase in the band intensity with no significant shift in peaks position indicating binding of APP IRE mRNA induces changes in the secondary structure alterations. Increased negative ellipticity of IRP1 in response to the addition of APP mRNA suggests gaining additional secondary structure. The addition of APP mRNA exhibited a change in ellipticity in the CD spectrum of IRP1 in the far-UV region, reflecting an increase in an α-helical content and a decrease in a β-sheet conformation upon binding to the APP mRNA. The percent secondary structure of free IRP1 and IRP1 bound to APP mRNA was calculated by the CDNN software. The secondary structural contents were estimated as described previously^40–42^. The binding of APP IRE mRNA to IRP1 increased the α-helix content to about 30% and reduced the β-sheet content to about 35%. We observed an increase in the α-helicity values of IRP1 with addition of APP IRE mRNA. These changes in the secondary structure reflect the IRP1 undergoing certain structural changes due to the addition of APP mRNA. This large structural alteration suggests IRP1 binds to APP mRNA by interacting with an α-helix, and that this conformational transition may plays role in kinetics of APP IRE mRNA with IRP1 complexes.

## Discussion

In this study we have investigated the detailed interaction between iron regulatory protein (IRP1) and stem-loop structure of IRE mRNA in the 5'-UTR of the APP transcript employing molecular docking, equilibria, kinetic, and circular dichroism. Previously^32^, we have shown that the IRP1 forms a complex with ferritin IRE, destabilizing this complex by iron. Here we employed previous knowledge from the ferritin IRE mRNA binding with IRP1 to extends the constraints of the known IRE stem-loop structure required to account for the iron-dependent translational regulation of the APP transcript.

Molecular docking revealed important amino acid residues Ser127, Arg269, Phe302, Thr438, Asn439, Ser475, Asn535, Arg728, Gly777, Ser778, and Ser780 of IRP1 that interact with APP IRE mRNA nucleotide. This molecular docking has validated our thermodynamic data that shows APP IRE mRNA∙IRP1 interaction is driven by the hydrogen bonding. The predicted structures (secondary, tertiary, and molecular docking model) were most energetically favorable. The stability of the complex formation depends on the conformation of the RNA and the protein molecule. CD analysis further confirmed the conformational change of the protein molecule with the addition of mRNA. Docking analysis of APP IRE mRNA∙IRP1 interaction shows that several amino acid residues are involved in hydrogen bonding. IRE mRNA binds inside the IRP1 pocket (Fig. 2C) by means of hydrogen bonding as the main driving force for these interactions.

Further, fluorescence studies revealed the complex formation between APP IRE mRNA and IRP1. The translation of 5'-UTR IRE mRNA expression increases in response to cellular iron levels, mediated by IRP1^43^. Iron increases the equilibrium dissociation of the APP IRE∙IRP1 complex. Within the studied temperature range, the binding of APP IRE mRNA to IRP1 is enthalpy-driven and entropy-favored. The negative value of Δ*G* shows that the reaction as spontaneous. The negative value of Δ*H* values suggest RNA-IRP1 interaction is hydrogen bond-driven and/or van der Waals forces. Adding iron to the APP IRE∙IRP1 complex significantly changes enthalpic and entropic contributions. The binding enthalpies comparison yielded an enthalpy change, resulting in IRP1∙APP IRE mRNA and IRP1∙APP IRE∙mRNA-Fe^2+^, equal − 15.7 kJ/mol. This enthalpic difference is comparable to the enthalpy change per hydrogen bond (Δ*H* = 13 kJ/mol)^44^. Our Δ*H* data was further supported by the Δ*G *data for the contribution of hydrogen bonding between APP IRE mRNA and IRP1 protein. Addition of Fe^2+^ (anaerobic) change Δ*G* value of ~ − 5.2 kJ/mol. This difference corresponds to the Δ*G* difference ~ 5–6 kJ/mol, which is expected value for a single hydrogen bond or a salt bridge formed between RNA and protein^36,45,46^. In the case of the ferritin IRE mRNA∙IRP1 complex, IRP1 contains two binding domains that form twenty-two bonds with the ferritin IRE mRNA^47^. The conversion of IRP1 from an aconitase to an IRE mRNA·IRP1 complex requires extensive conformation changes in the protein and the RNA^47^. However, our molecular docking data shows that IRP1 forms eleven hydrogen bonds with APP IRE mRNA. Comparison between the molecular docking and experimental data reveals that APP IRE mRNA interacts with IRP1 protein predominantly through a hydrogen bond. The conformational changes in the APP IRE mRNA·IRP1 complex may also be controlled thermodynamically, as reported previously for protein-RNA bindings (U1A protein binding to stem-loop II of U1 snRNA)^48^. These conformational changes most likely also play a role in the kinetics of the APP IRE mRNA association with IRP1.

Using stopped-flow fluorescence, we show the kinetic measurements of APP IRE mRNA binding to IRP1 protein in the absence and presence of iron. The kinetic mechanism is consistent with our previously determined reaction rates for the ferritin IRE mRNA with IRP1^37^. Equilibrium studies showed that APP IRE mRNA interacts strongly with IRP1. Addition of iron destabilized the binding of APP IRE to IRP1. To determine whether iron destabilized the complex by decreasing *k*_on_, increasing *k*_off_, or both, the kinetics of these interactions were measured. The rate constant for the binding of APP IRE mRNA with IRP1was somewhat comparable to the ferritin IRE mRNA binding to IRP1^37^. Our previous studies showed that the rate constant of the ferritin IRE mRNA binding to IRP1 was lowered sixfold with addition of iron^37^. At the same time, the rate constant has decreased ~ threefold of the APP IRE mRNA·IRP1 complex with the addition of iron.

Additionally, in the presence of iron, a faster APP IRE dissociation rate was observed from APP IRE·IRP1 complex. These data suggest that adding iron to the APP IRE mRNA·IRP1 complex results in slower binding properties and faster dissociation rates. Furthermore, we presented the detailed temperature-dependent binding rate of APP IRE mRNA binding with IRP1 in the absence and presence of iron, which was neither previously measured nor reported. Iron affect both the association and the dissociation rates for APP IRE binding to IRP1. A significant difference in the kinetic rates is seen with changes in temperature for the interaction of the APP IRE with IRP1 in the absence and presence of iron. The Arrhenius activation energies for the IRP1· APP IRE mRNA and IRP1∙APP IRE mRNA-Fe^2+^ were significantly different. The IRP1·APP IRE mRNA and IRP1∙APP IRE mRNA-Fe^2+^ had *E*_a_ of 52.5 ± 2.1 and 86.0 ± 3.2 kJ/mol, respectively. The addition of iron significantly changed the activation energy. The overall change in activation energy for APP IRE mRNA∙IRP1 complex with anaerobic addition of Fe^2+^ suggests that iron (II) succeeds in conformational change of the complex. Selective iron-induced conformational change of APP IRE·IRP1 complex emphasizes the sensitivity of RNA structure–function relationship to its environment. Our kinetic data is supported by thermodynamic equilibrium data for the contribution of conformational stability of the molecule, which was further confirmed by CD studies. Iron destabilizes the APP IRE·IRP1 complex through conformational changes produced on IRP1 protein upon APP IRE binding, similar to those observed for ferritin IRE mRNA binding to IRP1 and eIF4F^27^. The conformational change promotes a release of the repressor protein, IRP1, from the APP IRE·IRP1 complex, allowing eIF4F binding and increased translation of the APP mRNA. Our experiments show that APP IRE mRNA binding is accompanied by a conformational change in the IRP1 protein, resulting in changes in hydrogen bonds. The CD shows that the conformational change in IRP1 protein prompts increased secondary structure elements. The IRP1 protein structural change upon binding with APP IRE is significant. Conformational changes in the protein structure were also induced by the binding of small ligands, including IRE mRNA, inducing conformational changes^27,34^. The observed change in enthalpy and free energy supports the structural changes in the IRP1 protein upon binding to APP IRE mRNA.

The fact that the rate constant for the two IRE mRNA (ferritin *versus* APP) is similar for the IRP1 protein indicates that APP IRE also plays a role in iron regulation in the brain. Previously we have shown that iron facilitates the release of IRP1 from the ferritin IRE∙IRP1 complex, thus allowing the binding of eIF4F. This, subsequently, enhances ferritin mRNA translation^32,43,49^. A high iron level in the central nervous system has been observed in Alzheimer's patients^5^. Increasing cellular iron level lowers IRE mRNA∙IRP1 binding through structural alterations, and therefore increases its affinity with the initiation factors, and the ribosome assembly, thereby enhancing amyloid mRNA translation.

## Methods

### Preparation of RNA and protein

Human APP IRE mRNA oligonucleotide (57-nt) (sequence: -GGGGCCCCGGGAGACGGCGGCGGUGGCGGCGCGGGCAGAGCAAGGACGCGGCGG AUC) was purchased from Metabion International AG, Germany. After dissolving the RNA in 40 mM HEPES/KOH, pH 7.2, 100 mM KCl, and 5% glycerol. mRNA was melted and reannealed as described previously^32^ by heating to 85 °C for 15 min, followed by slow cooling to 25 °C. RNA concentration was quantified spectrophotometrically by measuring absorbance at 260 nm with a standard of 40 µg/ml RNA as 1. The purity of synthesized IRE mRNA was checked by measuring the absorbance ratio, having A_260/280 nm_, of 2 to 1. The IRP1 protein was purchased from OriGene, Rockville, MD, USA. The Bradford method^50^ measured protein concentration using Bio-Rad protein assay reagent with bovine serum albumin as a standard.

### 4.2. Molecular docking

The binding interactions of APP IRE mRNA oligonucleotide and IRP1 was explored by using the molecular docking approach. Structural coordinates of IRP1 were from the RCSB Protein Data Bank (PDB ID: 3SNP). Three-dimensional structural coordinates of APP IRE mRNA oligonucleotide were modelled through RNAComposer^30,51^ using the CentroidFold secondary structure prediction method^52,53^. The docking of APP IRE mRNA oligonucleotide with IRP1 was performed using the HDOCK server^31^ ADDIN EN.CITE^31,54^. First, the docking results were screened for higher binding affinity, and then top docked conformation was selected and analyzed using PyMOL and Discovery Studio Visualizer for their possible interactions.

### Steady-state fluorescence measurements

The binding of the APP IREmRNA to the IRP1 protein was measured by monitoring the change in intrinsic protein fluorescence intensity after addition of increasing amounts of IRE mRNA. Protein fluorescence was monitored at an excitation wavelength λ_Ex_ = 280 nm and an emission wavelength λ_Em_ = 332 nm. Binding studies of the IRP1 (0.1 µM) were performed with increasing amounts of the APP mRNA (0.0 – 350 nM) in 20 mM HEPES–KOH buffer, pH 7.4, containing 150 mM KCl and 1 mM MgCl_2_. When Fe^2+^ was used, all incubations were anaerobic. Anaerobiosis conditions of Fe^2+^ were maintained by purging all solutions with nitrogen and performed all reactions under nitrogen atmosphere. This prevents oxidation of ferrous iron to ferric state. Under such conditions, IRE mRNA is stable in the presence of Fe^2+^^32,43^. The change in fluorescence intensity of the protein titrated with APP mRNA was used to monitor RNA-IRP1 binding. Before the data collection, each sample was incubated for 15 min, allowing for equilibration to the experimental temperature. The sample temperature was maintained using a thermocouple device inside the cuvette (Δ*T* ± 0.1 °C) for all temperature-dependent binding experiments. The normalized fluorescence change (Δ*F*/Δ*F*_max_) of the IRP1·IRE mRNA complex and fluorescence spectra of the individual proteins were used to determine the equilibrium dissociation constant (*K*_D_), as described previously^27,55^. The fluorescence intensity of IRP1 protein (0.1 µM) alone was measured in the control experiments. Fluorescence intensity of the control sample was used to determine the corrected fluorescence intensity of the complex. Then, fluorescence intensity of another sample of APP mRNA at specific concentration was measured. When necessary, the protein fluorescence intensity data were corrected for the dilution and inner filter effects; maximum dilutions were < 5%. Samples were passed through a 0.22-µm filter to remove any suspended material. Three individual titration experiments were performed for all equilibrium measurements, and the average value was reported. KaleidaGraph (Synergy Abelbeck Software, version 2.1.3) was used to obtain the *K*_D_ values and standard errors as the mean ± SD.

### Thermodynamic measurements

To determine the thermodynamic parameters for the APP IREmRNA interaction with IRP1 protein in the absence and presence of iron, temperature-dependent binding constants were used to construct the van't Hoff plots. Thermodynamic parameters, the changes in enthalpy (Δ*H*), entropy (Δ*S*), and free energy (Δ*G*) of APP mRNA·IRP1 interactions are useful in characterizing the contribution of the interactive forces. Thermodynamic values were measured from the temperature dependence of the *K*_D_ according to the van't Hoff isobaric equation:

3 $$ln \; {K}_{a }= -\frac{\Delta H}{R T }+ \frac{\Delta S}{R}$$

where *R* is the gas constant (8.31 J mol^−1^ K^−1^) and *T* is the absolute temperature in Kelvins. *K*_a_ was determined at five different temperatures: 5, 10, 15, 20, and 25 °C, respectively. Δ*H* and Δ*S* values were determined from the slope and the intercept of ln *K*_a_ *versus* temperature (*T*^−1^) plot.

The change in free energy, Δ*G*, for the binding reaction between APP mRNA and IRP1 protein was calculated by the following equation:

4 $$\Delta G= \Delta H-T\Delta S \; and \; \Delta G= - R T \; ln \; {K}_{a}$$

where *T* is 298 K.

### Stopped-flow fluorescence measurements

Rapid kinetic measurements for the APP IREmRNA binding with the IRP1 protein were performed with a stopped-flow spectrometer. Fluorescence intensity (measured in volts) for the IRP1 was monitored at the cut-on filter of 324 nm with an excitation wavelength of 280 nm. Fluorescence intensity was monitored for up to 200 ms. The dead time of the instrument was 1 ms. About a thousand pairs of data points were collected in each sample reaction. The samples were incubated for 15 min to allow equilibration to the experimental temperature before the data collection. The sample was thermo-stated, and the temperature of the flow-cell reservoir was maintained with a temperature-controlled circulating water bath (Δ*T* ± 0.1 °C). After rapid mixing of IRP (0.1 μM, final) with APP mRNA (0.1, 0.2, 0.5, and 1.0 μM, final), the time course of the fluorescence intensity was recorded by computer data acquisition software. The stopped-flow traces from the three-to-five individual shots were averaged to optimize the signal-to-noise ratio. As described previously^49,55,56^, data were evaluated by fitting into the single- and double-exponential functions. We further observed the effects of 50 μM Fe^2+^ on the binding rates of the APP mRNA (0.1, 0.2, 0.5, and 1.0 μM final) with the IRP (0.1 μM final); the experiment was carried out under the same conditions as described above.

### Measurements of dissociation rate constants

To measure the dissociation rate constants of the pre-formed APP IRE mRNA·IRP1 complexes, the APP mRNA and the IRP1 protein samples were incubated at 25 °C in a titration buffer for 15 min to ensure complex equilibrium. The APP mRNA and the IRP1 protein concentrations in the reaction sample were 1.0 μM and 0.1 μM, respectively, after mixing. Dissociation rate constant of pre-formed APP mRNA·IRP1 complex was determined by monitoring the increase in fluorescence intensity signal of the IRP1 protein when equal volumes of the APP mRNA·IRP1 complex in one syringe and buffer alone, or buffer containing 50 μM Fe^2+^ in the second syringe, were mixed in the stopped-flow cell. The dissociation rates were determined for the relaxation experiment from the fits of the non-linear analytical equation using KaleidaGraph software (version 2.1.3), as described previously^46,57^.

### Kinetic data analysis and data fitting

Stopped-flow fluorescence traces representing binding of the APP mRNA with the IRP1 were analyzed according to a curve-fitting program (Global analysis software), as described previously^49,56^. Data from the fluorescence intensity (measured in volts) experiments were fitted to the single- and double-exponential functions. Fitted curves correspond to the following single exponential equation,

5 $${F}_{t}=R\times {e}^{\left(-{k}_{obs}\times t\right)}+{F}_{f}$$

where *k*_obs_ and *R* are the observed first-order rate constant and the amplitude, respectively.* F*_*f*_, is the final value of fluorescence, and* F*_t_ is the anytime observed fluorescence, *t*. Fitted curve correspond to the following double-exponential equation,

6 $${F}_{t}= {R}_{1}\times {e}^{(-{k}_{{obs}_{1}}\times t)}+ {R}_{2}\times {e}^{(-{k}_{{obs}_{2}}\times t)}+{F}_{f}$$

where *R*_1_ and *R*_*2*_ are the amplitudes for the first and second components of the reaction with observed rate constants *k*_obs1_ and *k*_obs2_, respectively. The subscripts 1 and 2 refer to the fast and slow phases. Each fit was assessed from the residuals, measuring the difference between the calculated fit and the experimental data. The relaxation experiments further analyzed the observed rate constants to determine *k*_on_ and *k*_off_ from *k*_obs_ = *k*_on_ [APP mRNA] + *k*_off_. A plot of *k*_obs_ *versus* APP mRNA concentrations was used to determine the *k*_on_ and *k*_off_ from the slope and the intercept of the plot. For dilution experiments of the APP mRNA·IRP1 complex, the *k*_off_ value was obtained from the previously determined *K*_eq_ values and using *K*_d_ = *k*_off_/*k*_on_ that gives *k*_obs_ = *k*_off_ ([APP mRNA]/*K*_d_ + 1). KaleidaGraph software (Version 2.1.3, Abelbeck software) was used for least-square fitting with linear equations and determining standard errors for parameters obtained from the fits. The constant rate values were represented as the mean, and standard deviation (± SD).

Furthermore, we measured the temperature-dependent (5, 10, 15, 20, and 25 °C) kinetic rates for binding the APP mRNA with the IRP1 protein. The temperature-dependent observed rate constant values were used to determine the activation energy of the APP IREmRNA∙IRP1 complexes in the absence and presence of 50 μM iron. The observed rate constants were used to construct the Arrhenius plots according to the following relations:

7 $$ln\,{k}_{obs }= -\frac{{E}_{a}}{R T }+ln\,A$$

where *E*_a_ is the activation energy, *k*_obs_ is the observed rate constant, *R* (8.314 J/K. mol) is the universal gas constant, *T* (Kelvin) is the absolute temperature, and *A* is the Arrhenius pre-exponential factor. The values of the activation energies were calculated using the slopes of the fitted linear plot of ln *k*_obs_ *versus T*^−1^.

### Circular dichroism (CD) spectroscopy

CD measurements were performed in the far-UV region on a Chirascan Plus spectropolarimeter (Applied Photophysics Ltd, UK), equipped with a thermostatically controlled circulating water bath with a cell holder under constant nitrogen flow. The instrument was calibrated with (*D*)-(+)-10-camphorsulfonic acid according to the procedures outlined by the manufacturer. Far-UV CD spectra (200–250 nm) of the IRP1 protein (0.1 µM) with the addition of varying amounts of the APP IRE mRNA (0–10 µM) were obtained at the 25 °C temperature. Spectra were measured after incubating each protein sample in the absence and presence of the APP mRNA for 15 min at 25 °C. The protein samples for the CD measurements were dialyzed and filtered through a Millipore filter (0.45 µm) to remove any suspended material. Each spectrum was collected with a scan speed of 50 nm/min, a response time of 1 s, and a 0.5 mm path-length quartz cell. Each spectrum comprised of an average of three-to-five scans and high-frequency noise reduction was applied before the final CD spectra. For each spectrum collected, the contribution from the buffer blanks and buffer containing 0–10 µM APP mRNA, if applicable, was subtracted from the respective spectra. The change in secondary structure content of the IRP1 protein in the absence and presence of different amounts of the APP mRNA was calculated using CDNN software. Secondary structures were also estimated from the amino acid sequence by the predictive methods, as described previously^58–61^. The helical content of the protein was estimated from the mean residue ellipticity (MRE) at 222 nm using the following equation^41,62^:

8 $$\%\mathrm{\alpha }-\mathrm{helix }=\left(\frac{{\mathrm{MRE}}_{222\mathrm{nm}}-2340}{30300}\right) \times 100$$

## Data Availability

The datasets used and/or analyzed during the current study are available from the corresponding author on reasonable request. All relevant data are within the manuscript.
